# Modelling the Evolutionary Dynamics of Viruses within Their Hosts: A Case Study Using High-Throughput Sequencing

**DOI:** 10.1371/journal.ppat.1002654

**Published:** 2012-04-19

**Authors:** Frédéric Fabre, Josselin Montarry, Jérôme Coville, Rachid Senoussi, Vincent Simon, Benoît Moury

**Affiliations:** 1 INRA, UR0407 Pathologie Végétale, Montfavet, France; 2 INRA, UMR1349 IGEPP (Institute of Genetics, Environment and Plant Protection), Le Rheu, France; 3 INRA, UR546 Biostatistique et Processus Spatiaux, Montfavet, France; University of Kentucky, United States of America

## Abstract

Uncovering how natural selection and genetic drift shape the evolutionary dynamics of virus populations within their hosts can pave the way to a better understanding of virus emergence. Mathematical models already play a leading role in these studies and are intended to predict future emergences. Here, using high-throughput sequencing, we analyzed the within-host population dynamics of four *Potato virus Y* (PVY) variants differing at most by two substitutions involved in pathogenicity properties. Model selection procedures were used to compare experimental results to six hypotheses regarding competitiveness and intensity of genetic drift experienced by viruses during host plant colonization. Results indicated that the frequencies of variants were well described using Lotka-Volterra models where the competition coefficients *β_ij_* exerted by variant *j* on variant *i* are equal to their fitness ratio, *r_j_*/*r_i_*. Statistical inference allowed the estimation of the effect of each mutation on fitness, revealing slight (s = −0.45%) and high (s = −13.2%) fitness costs and a negative epistasis between them. Results also indicated that only 1 to 4 infectious units initiated the population of one apical leaf. The between-host variances of the variant frequencies were described using Dirichlet-multinomial distributions whose scale parameters, closely related to the fixation index *F*_ST_, were shown to vary with time. The genetic differentiation of virus populations among plants increased from 0 to 10 days post-inoculation and then decreased until 35 days. Overall, this study showed that mathematical models can accurately describe both selection and genetic drift processes shaping the evolutionary dynamics of viruses within their hosts.

## Author Summary

Natural selection and genetic drift drive the evolution of virus populations within their hosts and therefore influence strongly virus emergences. To help predict future virus emergences, we developed a model that estimates simultaneously genetic drift and selection intensities using high-throughput sequence data representing the within-host population dynamics of *Potato virus Y* variants differing at most by two substitutions involved in pathogenicity properties. The competitiveness costs induced by these mutations as well as the mathematical expressions of the competition coefficients of virus variants were derived from Lotka–Volterra equations. High genetic differentiation of virus populations between hosts was evidenced as well as its hump-shaped behaviour with time. The modelling framework proposed here was intended to help design control strategies aiming to prevent virus emergences.

## Introduction

Plant virus emergences represent near half of emerging plant infectious diseases [1] and often have detrimental consequences for food production. Emergences result from complex processes leading to novel virus-vector-plant-environment interactions [2], [3]. At the ecosystem level, numerous ecological factors, often related to changes in agricultural practices [3], favour emergence by impacting the very biology of viruses and vectors. At the molecular level, evolutionary factors allow viruses to jump host species barriers. As most viruses transferred to new hosts replicate poorly, the existence of already adapted variants within virus populations is often crucial to achieve a successful jump [4]. Though high mutation rates of RNA viruses favour the existence of already adapted variants, their dynamics in the reservoir hosts also depend on the strength of natural selection and genetic drift [4], [5]. Disentangling how selection and drift shape the evolutionary dynamics of viruses is therefore required to understand emergences [2], [5]. Mathematical models, which already play an important role in scrutinising the effects of such mechanisms, are also essential for estimating the likelihood of future emergences. Their scope of applications ranges from the management of drug-resistance in infectious diseases [6] to the achievement of durable plant resistance [7], [8].

Natural selection is a deterministic process by which the frequencies of the fittest variants in a given environment increase [9]. Selective effects among virus variants differing only by one or two point mutations can be very strong. Indeed, viruses with small genomes, including RNA and ssDNA viruses infecting animals, plants and bacteria, are characterized by a high mutational sensitivity. Non-lethal mutations reduce fitness by 10–13% on average [10]. Genetic drift is a stochastic process by which frequencies of virus variants change due to random sampling effects. Its strength is usually characterized by the effective population size (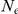) which is defined as the size of a theoretical population that would drift at the same rate as the observed population [11]. Although plant virus populations can reach extremely large sizes, estimates of 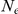 during colonization of plant tissues remained relatively small, ranging from units [12], [13] to a few hundreds [14]. These figures indicate that virus populations are often faced with narrow genetic bottlenecks that limit the fixation of advantageous mutations and allow slightly deleterious mutations to reach high frequencies [2].

While within-host genetic drift and selection act simultaneously, and thus jointly determine emergence, their intensities have rarely been estimated and modelled jointly from experimental data. Drift intensity was often measured using populations of pathogen variants with equal multiplicative fitness [14]–[16] and comparison of selection intensity acting on variants did not take into account genetic drift [17]–[19]. In the present work, we characterized experimentally and modelled the within-host population dynamics of four *Potato virus Y* (PVY) variants simultaneously submitted to genetic drift and natural selection. The four variants differ by one or two mutations that change their pathogenicity properties towards pepper genotypes carrying resistance alleles at a single locus [20]. Virus population dynamics were followed using high-throughput sequencing (HTS) [21] to track quantitatively the dynamics of PVY populations within a susceptible pepper host. Analysis of HTS data was performed with some sensible mathematical models which allowed inferring both the selection process between competing virus variants and the intensity of drift experienced by viruses during host plant colonization.

## Materials and Methods

### Plant and virus materials

**Figure 1:**
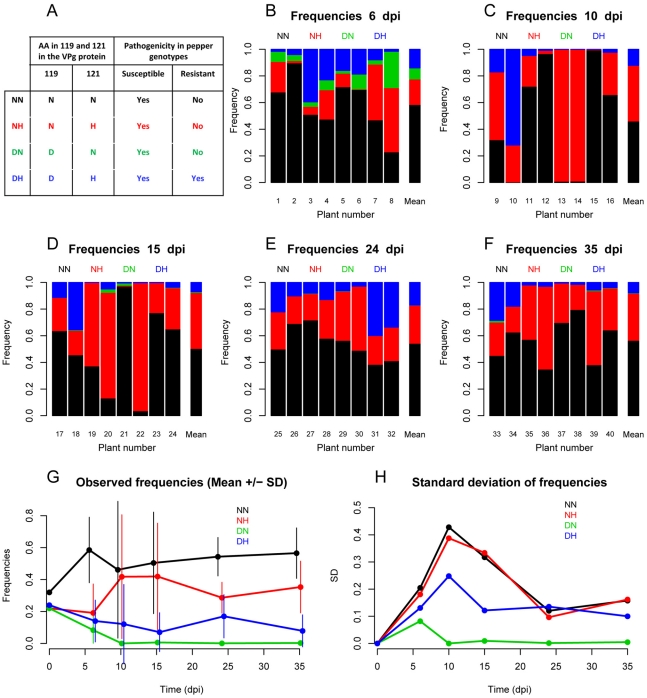
Observed intra-host dynamics of four PVY variants. **A**: Description of the four PVY variants used (NN, NH, DN and DH). Variants are named according to the amino acids at positions 119 and 121 of the VPg pathogenicity factor. All variants infect the pepper genotype Yolo Wonder (YW) but only DH infects the genotype Florida VR2 which carries the *pvr2^2^* resistance gene. **B–F**: Frequencies of the four PVY variants in the eight plant samples collected 6, 10, 15, 24 and 35 dpi. Additionally, for each date, a bar with the mean frequencies of the variants for the eight samples is provided. **G**: Mean (± standard deviation) frequencies of the four PVY variants as a function of time (dpi). **H**: Standard deviation of the frequencies of the four PVY variants as a function of time (dpi).

The pepper (*Capsicum annuum* L.) genotype used in this study was Yolo Wonder, a bell pepper cultivar susceptible to all PVY isolates. The SON41p infectious cDNA clone [22] and three derived PVY variants were used: NN, DN, NH and DH (the latter corresponding to SON41p). They were named after the amino acids observed at positions 119 and 121 of the VPg (viral protein genome-linked) pathogenicity factor (D, H and N representing aspartic acid, histidine and asparagine, respectively) (Figure 1A). The three mutated clones of SON41p differing by one or two substitutions in the VPg cistron were constructed using the QuikChange site-directed mutagenesis kit (Stratagene, La Jolla, CA, U.S.A.) [23]. Only variant DH, also termed resistance-breaking (RB) variant, was able to infect the pepper genotype Florida VR2 which carries the *pvr2^2^* resistance gene (Figure 1A) [20].

### Plant inoculation

Inoculations were carried out under insect-proof greenhouse conditions. First, separate inoculations with the cDNA clones were realized by DNA-coated tungsten particle bombardments of juvenile *Nicotiana clevelandii* plants (four week old) [22]. Crude extracts of infected *N. clevelandii* plants were calibrated using DAS-ELISA [23], adjusted by dilution, mixed equally and then inoculated mechanically on the two cotyledons of 40 Yolo Wonder plants approximately three weeks after sowing (*i.e.* at two-leaf stage). The conformity of each variant was checked by direct sequencing of the RT-PCR product corresponding to the entire VPg cistron of the PVY populations present in the four plants used for the inoculum [22].

### Sampling of PVY populations and HTS

Virus populations were separately sampled from eight plants at five successive dates: 6 days post-inoculation (dpi) corresponding to the 3–4 leaf stage, 10 dpi (5–6 leaf stage), 15 dpi (7–8 leaf stage), 24 dpi (11–12 leaf stage) and 35 dpi (22–23 leaf stage). Additionally, a sample of the mixed inoculum used for mechanical inoculations on pepper plants was also collected. At each date, all leaves of each plant were harvested, homogenized in a buffer (0.03 M phosphate buffer (pH 7.0) supplemented with 2% (w ∶ v) diethyldithiocarbamate; 4 mL of buffer per gram of leaves) and total RNAs were purified with the Tri Reagent kit (Molecular Research Center Inc., Cincinnati, OH, U.S.A.) from a 150 µL aliquot of each sample. RNAs were used to amplify the central part of the VPg cistron by RT-PCR with *Avian myeloblastosis virus* reverse transcriptase (Promega), the high-fidelity Herculase II fusion DNA polymerase (Stratagene) and primers PYRO-FOR (5′-attcatccaattcgttgatcc-3′, nucleotide positions 5930 to 5950) and PYRO-REV (5′-tgtcacaaaccttaagtggg-3′, nucleotide positions 6149 to 6168). Emulsion-PCR and high-throughput 454 sequencing were realized by GATC-Biotech (Konstanz, Germany). The genome region sequenced encompasses notably codons 101 to 123 of the VPg cistron which has been demonstrated by reverse genetics to be the only region involved in breakdown of the *pvr2* resistance genes in pepper [20]. Since sampling was destructive, virus populations at the successive dates came from different plants.

In addition, in order to estimate the effective population size during leaf colonization, the eight plants sampled at 15 dpi were kept till 50 dpi and then a single newly grown leaf was sampled randomly on each plant (Figure S1). These leaves were individually crushed, total RNAs were purified and HTS of the central part of the VPg cistron was performed as described above. In the analysis, the virus populations characterized at 15 dpi constituted the “initial” populations and the ones at 50 dpi the “final” populations. In this protocol, as explained in [14], the “initial” populations defined by sampling all infected leaves at 15 dpi is likely to represent best the virus population circulating within the vascular system as infected leaves at 15 dpi have previously received (and exported) viruses from (and into) the vascular system. The “final” populations, sampled in a single systemically infected and newly grown leaf, necessarily originate from the “initial” populations regardless of the many successive stages of the systemic infection. The estimation of the effective population size arose from the comparison between the genetic variances of the “initial” and “final” populations.

### Determination of PVY variant frequencies

We obtained between 209 and 930 correctly assigned sequences per virus population of each pepper plant, corresponding to a total of 24,166 sequences. Alignment was done using default parameters of the software SeqMan (DNASTAR Lasergene, Madison, U.S.A.). Because indels are the most frequent 454 pyrosequencing errors [24], a program, developed using the software R version 2.9.2 (R Development Core Team, 2009), was used to remove insertions and to replace deletions by the nucleotide present at the corresponding position in the four PVY variants. Because the program removed short sequences, the total number of cleaned sequences reduced to 20,795, ranging from 184 to 824 per virus population. Since no mutation with a significant frequency (>1%) has been observed in the sequenced region for any PVY population, the census of each variant (NN, DN, NH and DH) was determined for each sample according to the two polymorphic sites at codon positions 119 and 121 of the VPg coding region initially present in the PVY population (Table S1).

### Modelling within-host virus population dynamics

#### General model framework

To investigate the time course of competing virus populations within host plants, we merged deterministic and stochastic aspects into a single model. The deterministic aspect relied on the assumption that the mean numbers (or densities) *V_i_(t)* of virus variants *i = 1,…,4* at time *t*, within a host plant behaved as a deterministic interacting system of four ordinary differential equations (ODE):
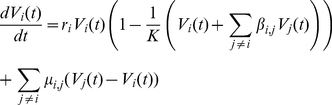
(1)Model parameters have the following interpretation:

- *K* is the virus carrying capacity of a plant,
- *r_i_* is the intrinsic rate of increase of variant *i*,
- *β_i,j_* is the coefficient that accounts for the competition strength exerted by genotype *j* on genotype *i*,
- *μ_i,j_* is the mutation rate from genotype *j* to genotype *i*.

Equation 1 extends Lotka-Volterra competition equations to four competitors [25] and introduces the mutation processes occurring between virus variants. We assumed that mutations occur independently. Thereby, if *μ* denotes the point mutation rate per replication cycle and per nucleotide, we get 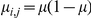 if variants *i* and *j* are distant from one mutation and 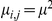 if variants *i* and *j* are distant from two mutations.

The stochastic aspect took account of random fluctuations from the theoretical means *V_i_(t)* due to the heterogeneity of virus populations between plants and to the nature of samples (counts of virus sequences). To be more specific, let 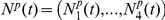 denote the vector of numbers of sequences observed for plant *p* sampled at time *t* (dpi) corresponding to virus variants NN, DN, NH and DH. Our second main assumption states that, conditionally to the total number 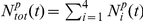 of sequences, the random vector 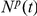 resulted from a Dirichlet-multinomial (DM) distribution, *i.e.*:
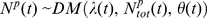
(2)where *(i)* 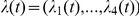 is the theoretic vector parameter of variant frequencies at time *t* over all plants (*i.e.* 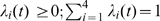 ) and

*(ii)* 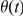 is a scale parameter related to the intensity of virus genetic differentiation between plants at time *t*.

For clarity, we recall that an integer valued vector *N* (discarding time and plant indices *t* and *p*) is 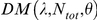 distributed if *N* results from a two-step random procedure. First, a non-observed random vector 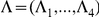 is drawn from a Dirichlet distribution 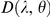. Then, given a total number *N_tot_* and random frequencies Λ, *N* is drawn according to a multinomial 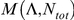. Our assumption finally reads as: sampling a plant *p* at time *t* gives rise to a non-observed random vector 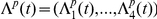 of variant frequencies depending on virus population differentiation *θ(t)* and on overall theoretic means λ*_i_(t)* of the variant frequencies. The numbers 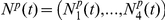
of observed sequences of virus variants in plant *p* at time *t* is a multinomial 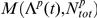. Since 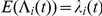 and 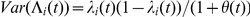, large values of *θ(t)* decrease the genetic differentiation among host plants. Asymptotically, if 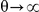, the Dirichlet multinomial distribution converges towards a simple multinomial distribution without genetic differentiation at all, *i.e.* Λ is deterministic and equals λ. Besides, *θ(t)* can be actually related to the so-called fixation index *F*_ST_ [11] by 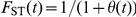 [26].

Finally, the coupling between deterministic and stochastic components consisted in asserting that the overall frequencies of variants at time *t* were given *via* the ODE system as 
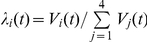
.

### Within-host virus dynamics: model selection and inferences

Our goal was to determine (i) the forms of selection processes occurring between competing viruses within a host plant, which could be handled *via* the forms of the competition coefficients used to derive the overall theoretical frequencies *λ_i_(t)* and (ii) the intensity and temporal variation of genetic drift experienced by viruses during host plant colonization, which could be investigated *via* the time dependence of the scale parameter *θ(t)*.

Accordingly, six models allowing four to 14 parameters (Table 1) were considered. All models included four intrinsic rates of increase 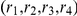 (under the constraint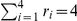). Regarding the competition issue, three embedded hypotheses were proposed for the Lotka-Volterra competition coefficients. From general to particular:

(C_1_) 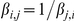: inverse reciprocal competition of virus variants without any prior,
(C_2_) 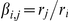: inverse reciprocal competition based on the ratio of intrinsic rates of increase,
(C_3_) 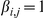: blind and uniform competition between virus variants.

**Table 1:** Models description and selection criteria.

Model^a^ (number of parameters)	Genetic differentiation between plants^b^	Virus selection within plants^c^	−2.log(L)^d^	AIC	BIC
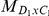 (14)	D_1_: 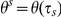	C_1_: 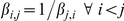	940	968	992
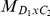 (8)	D_1_: 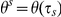	C_2_: 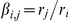	951	967	980
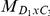 (8)	D_1_: 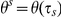	C_3_: 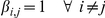	956	973	987
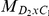 (10)	D_2_: 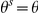	C_1_: 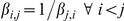	999	1019	1036
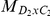 (4)	D_2_: 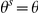	C_2_: 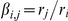	1016	1024	1031
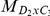 (4)	D_2_: 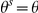	C_3_: 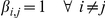	1001	1010	1017

a Six models are obtained by crossing 2 hypotheses regarding the genetic differentiation of virus populations between plants (D_1_ and D_2_) with 3 hypotheses regarding the competition issue between virus variants (C_1_ to C_3_). They include four to 14 parameters and were compared using Akaike information criterion (AIC) and Bayesian information criterion (BIC) to identify the model that is best supported by the data.

b The process of genetic differentiation of the virus populations between plants, described by the scale parameter *θ* of a Dirichlet-multinomial distribution, was allowed either to be constant (*θ^s^* = *θ* 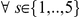) or time varying (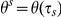, for the five sampling dates 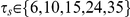).

c The process of virus competition within plants included the intrinsic rates of variant increase 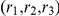 (given that 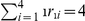) as parameters but might undergo one of three hypotheses specifying the type of Lotka-Volterra competition coefficients 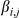.

d −2log(Likelihood).

Regarding the genetic differentiation of virus populations between plants, two embedded hypotheses were considered. From general to particular:

(D_1_) 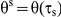 where s = 1,‥,5 are free parameters: genetic differentiation between plants is time dependent (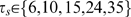 in dpi),
(D_2_) 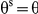 where s = 1,‥,5: genetic differentiation between plants is constant with time.

Six models, denoted 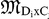 (Table 1), are obtained by crossing hypotheses D_i_ with C_j_. Under the constraint 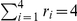 and by setting K to 10^6^ and *μ* to 10^−5^ [27], the six models were statistically identifiable using maximum likelihood techniques. For initial values 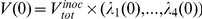 of ODE system, 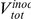 was arbitrarily set to 100 whereas 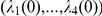 = (0.32, 0.22, 0.22, 0.24) corresponded to the observed frequencies of virus sequences in the inoculum. A note on model identifiability and likelihood-based inferences is provided in Text S1. Computations were performed with the R software environment using “bbmle” package and “nlminb” optimization routines. Models were compared using AIC and BIC procedures (Akaike and Bayesian Information Criteria) to choose the model that is best supported by the data.

### Estimation of the effective virus population size during host colonization

In order to estimate the effective population size (*N_e_*), *i.e.* the number of founder infectious units initiating the systemic infection of a single leaf, we used a method based on *F*_ST_ statistics described in [14]. Population genetics theory asserted that, for a haploid organism, 
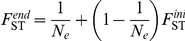
, where 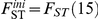 (resp. 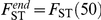) corresponds to *F*_ST_ value at 15 dpi (resp. 50 dpi). A 95% confidence interval was calculated for *N_e_* with a bootstrap method by resampling data 1,000 times over plants.

## Results

### Intra-host virus dynamics: insights from raw data

HTS of the composite inoculum confirmed that DAS-ELISA used to calibrate PVY variants concentration was accurate. The frequencies observed *a posteriori*, *i.e.* 0.32, 0.22, 0.22 and 0.24 for the NN, DN, NH and DH variants, respectively, were quite close to the expected frequency of 0.25, although an excess of NN was noticed.

During the course of the experiment, variant NN was selected; its mean frequency increased from 0.32 in inoculum to 0.57 in the populations sampled at 35 dpi (Figure 1G). This selection took place rapidly and could be detected as soon as at 6 dpi. Selection was also observed for variant NH, whose mean frequency increased from 0.22 to 0.35. Conversely, variants DN and DH were counter-selected: their mean frequency decreased from 0.22 (resp. 0.24) in the inoculum to 0.003 (resp. 0.07) at 35 dpi (Figure 1G).

Besides these mean trends, the standard deviation of variant frequencies between plants exhibited remarkable dynamics (Figures 1B to 1F, Figure 1H). It reached a maximum at 10 dpi, except for DN which had the lowest frequency. In four out of the eight plants analyzed at 10 dpi, a single variant (NH in two plants and NN in two others) dominated (Figure 1C). This observation indicated that virus populations underwent strong stochastic variations until 10 dpi. Later, the genetic differentiation of virus populations between plants tended to decrease: two weeks later, at 24 dpi, three variants (NN, NH, DH) co-infected all the eight plants analyzed (Figure 1E).

### Intra-host virus dynamics: insights from data modelling

**Figure 2:**
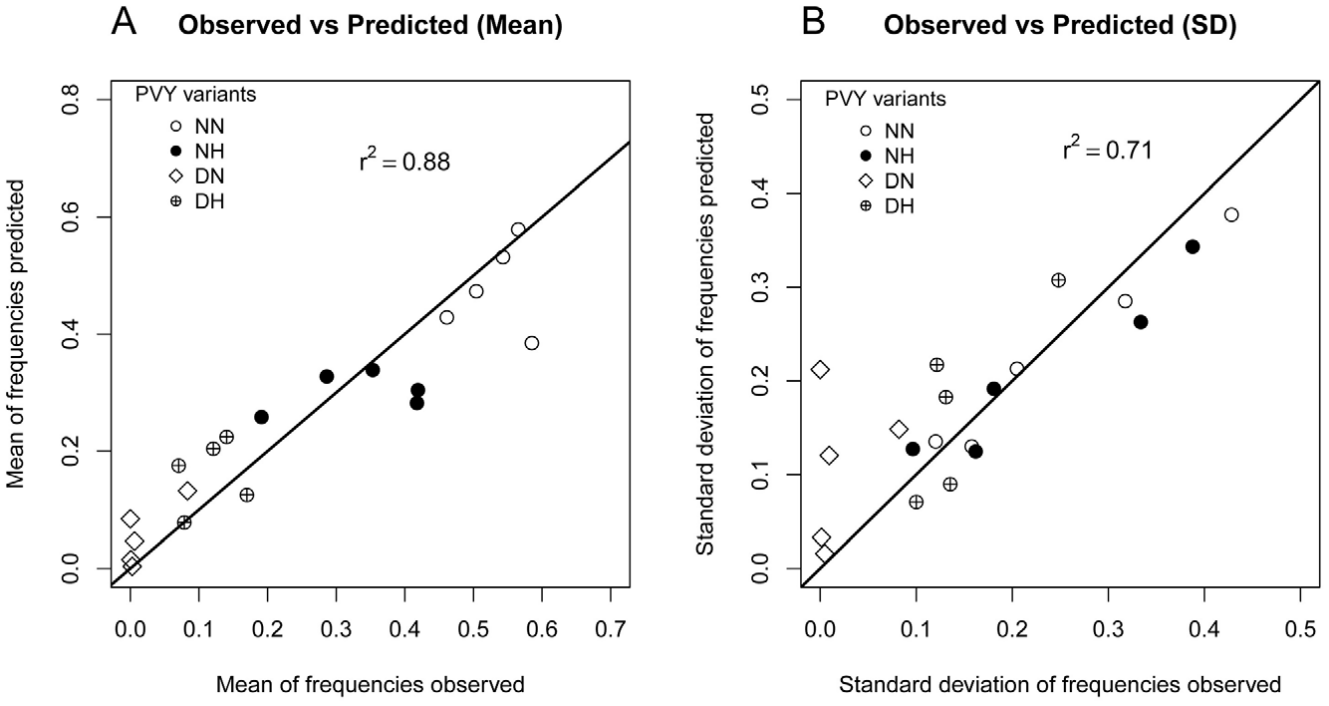
Goodness of fit of the model that is best supported by the data (model 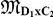
 **).** **A**: Correlation between the 20 (4 variants×5 dates) observed mean frequencies of the four PVY variants and their estimated mean values. **B**: Correlation between the 20 observed standard deviations of the frequencies of the four PVY variants and their estimated standard deviations. The full line is the first diagonal (*i.e.* line *y* = *x*).

A model selection procedure was used to test hypotheses concerning the selection process occurring between competing virus variants within a host plant and the intensity of drift experienced by viruses during host plant colonization. According to both AIC and BIC criteria, the model 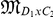 (Table 1) was best supported by the data. It satisfactorily fitted the data with an *r^2^* value of 0.88 between observed and predicted mean frequencies of virus variants (Figure 2A) and of 0.71 between observed and predicted standard deviations of these frequencies (Figure 2B). The root mean square error (RMSE) was 0.07. Inference was not sensitive (percentage of variations <5%) to 1000 fold ranges of variation of the mutation rate *μ* and of the number of inoculated viruses 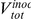. Inference was also only slightly sensitive to a 20% random fluctuation of the inoculum initial frequencies (Text S1).

**Figure 3:**
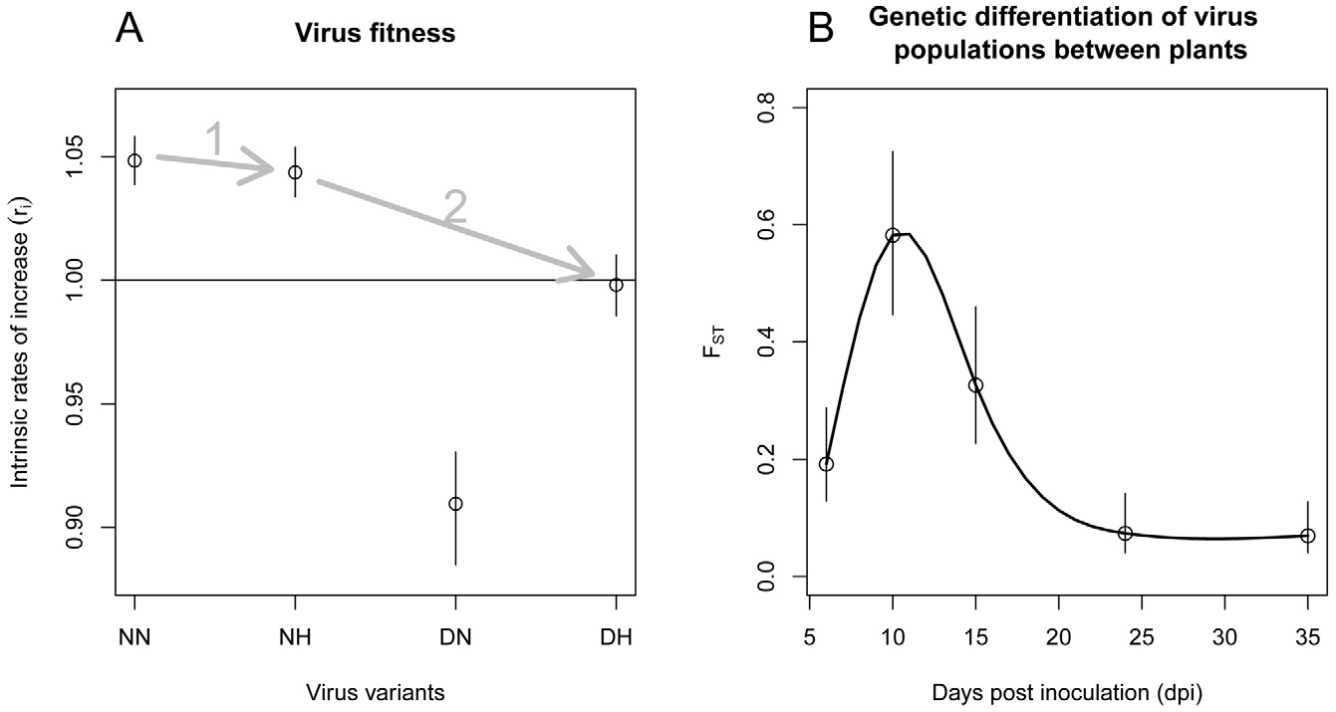
Parameter estimates of the model that is best supported by the data (model 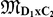
 **).** **A**: Relative fitness of the four PVY variants (NN, NH, DN and DH) estimated by their intrinsic rates of increase *r*. The mean fitness of the population was arbitrarily set to 1 due to identifiability constraints (Text S1). In both graphs, dots indicate the mean values of the parameter whereas segments stand for the 95% confidence interval. Arrows indicate the most likely pathway leading to the resistance-breaking variant. **B**: *F*_ST_ indices as a function of time (dpi). *F*_ST_ characterizes the degree of genetic differentiation of the virus populations between plants. For each sampling date 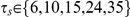, 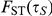 was assessed as 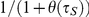 where 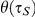 is the scale parameter of a Dirichlet-multinomial distribution. For illustration purposes, a spline function (full line) is fitted to data.

Inference on intrinsic rates of increase revealed significant differences of fitness between the four variants (Figure 3A). Variants NN and NH had the highest increase rates (1.048 for NN and 1.043 for NH), significantly higher than the one of DH (0.99). Variant DN had significantly the lowest rate of increase (0.91). Moreover, the selection of the model 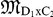 lent support to Lotka-Volterra competition coefficients 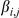 expressed as the ratio 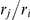.

Regarding the intensity of drift experienced by viruses during plant colonization, selection of model 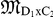 indicated that the genetic differentiation of virus populations between plants varied significantly with time. So did the fixation indices, *F*_ST_ (Figure 3B). The differentiation was maximal at 10 dpi, with an *F*_ST_ of 0.58 and minimal at 35 dpi with an *F*_ST_ of 0.069.

### Stable equilibrium of the Lotka-Volterra system

The long term behaviour of the model that is best supported by the data (Lotka-Volterra system with 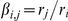 and mutation process) was theoretically studied. The differential system admits a single stable equilibrium which attracts all possible trajectories (*i.e.* the equilibrium point does not depend on initial conditions). The detailed proof is provided in Text S2. At equilibrium, all variants co-exist but no simple analytical expression of the equilibrium could be derived. Analytical results showed that the fittest variant predominates at equilibrium point whereas the density of other variants depended largely on (i) their genetic distance from the fittest variant and (ii) the difference between the intrinsic increase rate of the fittest variant and their own increase rate. According to parameter estimates, the relative frequencies of variants NN, DN, NH and DH at equilibrium were 0.9978, 0.0021, 7.18×10^−5^ and 4.34×10^−7^, respectively.

### Estimation of *N_e_* during plant colonization

*N_e_* during plant colonization was estimated by comparing the genetic variances of PVY populations sampled at 15 dpi (initial populations) and at 50 dpi (final populations) among the same plants. At 15 dpi, three variants were systematically detected in all plants, although with varying frequencies (Figure 1D). The final PVY populations, sampled from a single apical leaf of each plant at 50 dpi, differed largely from the initial ones. For seven out of eight plants, the frequency of variant NN was >0.85, while in the remaining plant, variant NH predominated (Table S1). This observation supported the hypothesis of large stochastic variations during the systemic infection of a newly formed leaf. Accordingly, the estimated effective population size 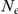 amounted to 2.25 (Table S2) with a 95% confidence interval ranging from 1.3 to 3.38. Actually, the method did not allow to disentangle the effects of selection and genetic drift on 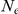. When applied only to the two variants NN and NH showing equal relative fitness (Figure 3A) and therefore subjected only to genetic drift, *N_e_* was estimated to 2.14 with a 95% confidence interval ranging from 1.29 to 3.41.

## Discussion

The present study investigated with HTS the intra-host dynamics of plant virus populations and their variability between plants. Data revealed a strong pattern of genetic differentiation of virus populations between plants with *F*_ST_ indices that increased from inoculation date to 10 dpi and then decreased until 35 dpi. From inoculation to 6 dpi, heterogeneity observed between plants could be potentially related to the very inoculation process and/or to the process of colonization of plants by viruses. Although we cannot exclude some random effects due to inoculation, we believe that most observed variance was due to within-plant colonization processes, as the four variants inoculated were detected still in all samples at 6 dpi (Figure 1B). Severe bottlenecks act on most virus populations at all the scales within infected plants, from virus loading into individual cells (MOI, multiplicity of infection, defined as the number of virus genomes that enter and effectively replicate in individual cells [15], [28]), to colonization of tissues and organs through plasmodesmata [29] and to translocation in the whole plant through the vascular system [12], [13], [30]. We strengthen the latter results for another RNA virus by showing that between one to four PVY genomes initiate the population of systemically-infected leaves. However, severe bottlenecks during host colonization are not necessarily the rule for all plant viruses. Using the same protocol, several hundred genomes of CaMV, a DNA virus, were shown to initiate infection of apical leaves [14].

The scale of our study was the set of leaves of infected plants. It represents the epidemiologically-relevant part of the virus population since it is readily accessible to vectors that ensure plant-to-plant horizontal transmission. It is possible that the narrow bottlenecks and intense genetic drift observed at larger scales (whole plant and plant organs) are direct consequences of those incurred by virus populations at the smallest scale (individual cell). Supporting this hypothesis, the time dependence of *F*_ST_ which we observed at the whole-plant level (Figure 3B) parallels that of the MOI in another plant virus [15]. MOI values observed by [15] were close to 2 at 14 dpi, increased up to 13 at 40 dpi and then decreased to the initial level at 70 dpi. Accordingly, we observed a *F*_ST_ decrease in the time period shared by both studies (from 14 to 35 dpi).

It is also possible that decreasing *F*_ST_ values observed at later infection times are related to the sink-source transition undergone by leaves during plant growth. Source-to-sink translocation of carbohydrates through the phloem corresponds to the direction of the systemic movement of viruses within plants [31], [32]. As the plant grows, more and more leaves behave as virus sources, a process beginning in oldest leaves. Consequently, as the plant matures, more leaves unload their viruses into the phloem sieve elements and can contribute to the colonization of new expanding leaves at the apex of the plant, hence increasing the size of the source virus population within plants and decreasing between-plant *F*_ST_ values. In the context of our experiments, the timing of the sink-source transition in pepper and its comparison with *F*_ST_ variation remain to be determined. The two above hypotheses are not mutually exclusive, since the increasing number of source leaves during infection could also increase the MOI [15], and, in turn, decrease between-plant *F*_ST_ values.

Even if major stochastic events impact the evolutionary dynamics of virus populations, natural selection remains a powerful force in virus evolution [2]. In our experiment, two variants were selected (NN and NH) and the other two counter-selected (DN and DH). The fitness effect of each mutation can be assessed from their intrinsic rates of increase. Compared to the fittest variant (NN), the fitness effects of mutations N_121_H and N_119_D amounted to −0.45% and −13.2%, respectively. These figures agree with the distribution of the mutational effect of single nucleotide substitutions. Indeed non-lethal mutations reduce virus fitness by 10–13% on average [10]. The fitness cost (4.8%) of the variant combining both mutations (DH) indicated a case of negative epistasis, as often observed for RNA viruses [2], [4]. Altogether, these data determined the most likely evolutionary pathway toward the breakdown of the pepper resistance gene *pvr2^2^* (mutation N_121_H followed by mutation N_119_D) [4], [33] (Figure 3A). In all, the RB variant is counter-selected in virus populations. When extrapolating the dynamics of the Lotka-Volterra system, the mean frequency of the RB variant would be <4% at 50 dpi. This prediction fitted our observation of ∼0.5% mean frequency of the RB variant at 50 dpi, although these results should be read with caution, as the sampling scheme differed at this date. Moreover, even if the long-time behaviour of the system indicated that all variants would co-exist at an equilibrium (which corresponds to the mutation-selection balance), the frequency of the RB variant would be very low (∼5×10^−7^). In natural context, the RB variant would most likely appear initially by mutation at a very low frequency in a virus population largely dominated by the fittest variants NN and NH. Note also that, although multidrug-resistant variants can be generated by recombination (*e.g.* [34] on HIV), its role is unlikely in the present case because the two critical amino acid positions are only two amino acids apart. Altogether these data provide an explanation of the scarcity of viruses able to overcome the resistance gene *pvr2^2^* in natural context [20].

By confronting the results of virus dynamics simulated under several Lotka-Volterra models differing by the form of the competition coefficients, we learnt about the selection process occurring between competing virus variants. These coefficients describe the interactions of several competing variants for host factors necessary for virus replication and movement within plants. Statistical model selection results lent support to the hypothesis that the competition coefficients *β_ij_* exerted by variant *j* on variant *i* are equal to their fitness ratio, 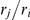. The assumption 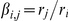 was initially argued on a theoretical ground [35]. These authors showed that Eigen's model of molecular quasispecies [36] was to a large extent equivalent to the Lotka-Volterra competition equations when assuming that 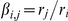. However this did not imply that the virus populations studied behaved as quasispecies. In particular, the quasispecies model does not allow for stochastic changes in population structure [30] whereas, as discussed earlier, the present results evidenced strong effects of genetic drift.

Overall, this study showed that mathematical models can accurately describe both selection and genetic drift shaping the evolutionary dynamics of viruses within hosts. A similar within-host model was coupled with an epidemiological model in [37] to assess the relative effects of ten demo-genetic and epidemiological parameters on the probability of breakdown of a plant resistance. Our present results validated *a posteriori* this choice. More generally, the modelling framework proposed here might provide a valuable cornerstone of models linking within- and between-host scales of disease dynamics [38]. It also might provide useful tools to study the interplay between the evolutionary and epidemiological processes acting on a virus population, at the individual host scale but also at the population host scale [39], and ultimately to design some efficient control strategies of virus emergence.

## Supporting Information

Figure S1 Protocol used to estimate the effective population size during the colonization of a pepper leaf by *Potato virus Y*. The initial (all leaves at 15 dpi) and final (a single apical leaf chosen randomly at 50 dpi) virus populations are represented in blue and red, respectively.
(PDF)

Table S1 Number of well-assigned and cleaned sequences to each sample (eight plants by date and the mixed inoculum) and number and frequency of each virus variant (NN, NH, DN, and DH) in each population.
(PDF)

Table S2 Estimation of *N_e_* during plant colonization.
(PDF)

Text S1 Note on model identifiability and on likelihood-based inference.
(PDF)

Text S2 Mathematical analysis of the Lotka-Volterra model with mutation.
(PDF)
