# Supplementary material for: Modelling the Evolutionary Dynamics of Viruses within Their Hosts: A Case Study Using High-Throughput Sequencing
Source: PLoS Pathog. 2012 Apr 19;8(4):e1002654. doi: 10.1371/journal.ppat.1002654 (PMC3330117; doi:10.1371/journal.ppat.1002654)

Figure S1. Protocol used to estimate the effective population size during the colonization of a pepper leaf by *Potato virus Y*. The initial (all leaves at 15 dpi) and final (a single apical leaf randomly chosen at 50 dpi) viral populations are represented in blue and in red, respectively.

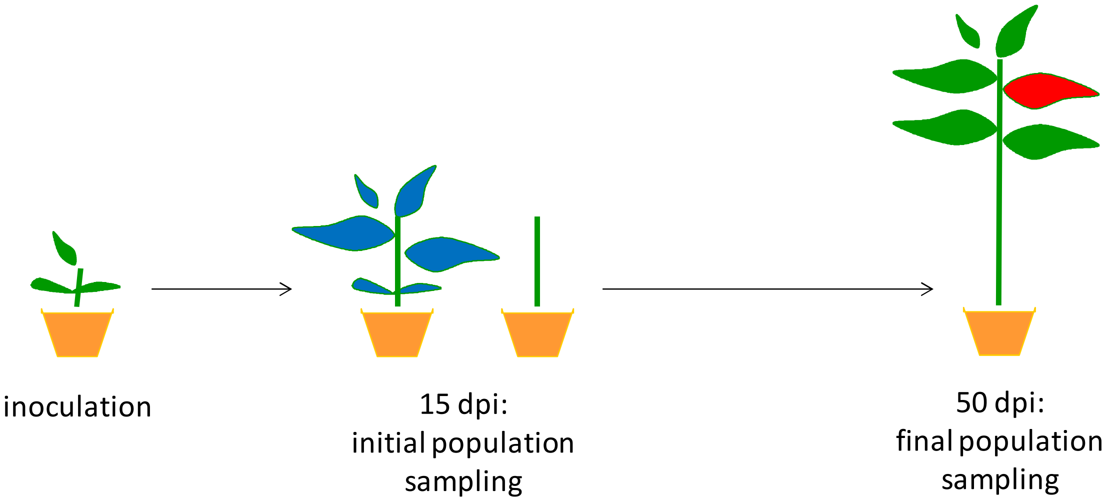

Supplement: Figure S1 — Protocol used to estimate the effective population size during the colonization of a pepper leaf by Potato virus Y. The initial (all leaves at 15 dpi) and final (a single apical leaf chosen randomly at 50 dpi) virus populations are represented in blue and red, respectively. (PDF) [file ppat.1002654.s001.pdf]
