# Supplementary material for: Modelling the Evolutionary Dynamics of Viruses within Their Hosts: A Case Study Using High-Throughput Sequencing
Source: PLoS Pathog. 2012 Apr 19;8(4):e1002654. doi: 10.1371/journal.ppat.1002654 (PMC3330117; doi:10.1371/journal.ppat.1002654)
Supplement: Table S1 — Number of well-assigned and cleaned sequences to each sample (eight plants by date and the mixed inoculum) and number and frequency of each virus variant (NN, NH, DN, and DH) in each population. (PDF) [file ppat.1002654.s002.pdf]

Table S1. Number of well assigned and cleaned sequences to each sample (eight plants by date and the mixed inoculum) and number and frequency of each viral variant (NN, DN, NH and DH) in each population.

				Number of cleaned sequences for each PVY variants				Relative frequencies of each PVY variants			
**Sample**	**dpi**	**Assigned sequences**	**Cleaned sequences**	**NN**	**DN**	**NH**	**DH**	**NN**	**DN**	**NH**	**DH**
**1**	**6 dpi**	209	184	125	14	42	3	0.679	0.076	0.228	0.016
**2**	**6 dpi**	523	373	335	17	6	15	0.898	0.046	0.016	0.040
**3**	**6 dpi**	565	471	241	16	28	186	0.512	0.034	0.059	0.395
**4**	**6 dpi**	357	309	147	23	68	71	0.476	0.074	0.220	0.230
**5**	**6 dpi**	570	483	347	10	49	77	0.718	0.021	0.101	0.159
**6**	**6 dpi**	295	225	157	25	1	42	0.698	0.111	0.004	0.187
**7**	**6 dpi**	745	653	307	21	273	52	0.470	0.032	0.418	0.080
**8**	**6 dpi**	444	378	87	103	182	6	0.230	0.272	0.481	0.016
**9**	**10 dpi**	298	252	81	0	128	43	0.321	0.000	0.508	0.171
**10**	**10 dpi**	826	714	3	0	198	513	0.004	0.000	0.277	0.718
**11**	**10 dpi**	366	317	229	0	73	15	0.722	0.000	0.230	0.047
**12**	**10 dpi**	312	245	237	0	6	2	0.967	0.000	0.024	0.008
**13**	**10 dpi**	466	419	4	0	415	0	0.010	0.000	0.990	0.000
**14**	**10 dpi**	930	824	9	0	815	0	0.011	0.000	0.989	0.000
**15**	**10 dpi**	466	402	400	0	2	0	0.995	0.000	0.005	0.000
**16**	**10 dpi**	766	655	432	0	208	15	0.660	0.000	0.318	0.023
**17**	**15 dpi**	602	522	333	0	130	59	0.638	0.000	0.249	0.113
**18**	**15 dpi**	545	464	212	2	85	165	0.457	0.004	0.183	0.356
**19**	**15 dpi**	436	383	143	0	240	0	0.373	0.000	0.627	0.000
**20**	**15 dpi**	274	241	32	6	191	12	0.133	0.025	0.793	0.050
**21**	**15 dpi**	515	429	417	7	3	2	0.972	0.016	0.007	0.005
**22**	**15 dpi**	466	432	16	0	415	1	0.037	0.000	0.961	0.002
**23**	**15 dpi**	735	625	483	0	141	1	0.773	0.000	0.226	0.002
**24**	**15 dpi**	699	599	390	2	185	22	0.651	0.003	0.309	0.037
**25**	**25 dpi**	332	298	149	0	83	66	0.500	0.000	0.279	0.221
**26**	**25 dpi**	309	266	184	0	55	27	0.692	0.000	0.207	0.102
**27**	**25 dpi**	431	369	265	1	73	30	0.718	0.003	0.198	0.081
**28**	**25 dpi**	239	210	122	0	61	27	0.581	0.000	0.290	0.129
**29**	**25 dpi**	357	315	178	1	116	20	0.565	0.003	0.368	0.063
**30**	**25 dpi**	259	214	105	0	103	6	0.491	0.000	0.481	0.028
**31**	**25 dpi**	663	589	227	0	127	235	0.385	0.000	0.216	0.399
**32**	**25 dpi**	739	652	269	0	164	219	0.413	0.000	0.252	0.336
**33**	**35 dpi**	256	228	103	3	57	65	0.452	0.013	0.250	0.285
**34**	**35 dpi**	411	341	214	0	66	61	0.628	0.000	0.194	0.179
**35**	**35 dpi**	471	407	233	0	166	8	0.572	0.000	0.408	0.020
**36**	**35 dpi**	456	412	144	0	256	12	0.350	0.000	0.621	0.029
**37**	**35 dpi**	484	415	290	0	123	2	0.699	0.000	0.296	0.005
**38**	**35 dpi**	444	394	314	0	74	6	0.797	0.000	0.188	0.015
**39**	**35 dpi**	391	332	127	2	184	19	0.383	0.006	0.554	0.057
**40**	**35 dpi**	434	381	245	1	120	15	0.643	0.003	0.315	0.039
**41**	**50 dpi**	410	354	351	0	1	2	0.992	0.000	0.003	0.006
**42**	**50 dpi**	305	257	255	0	2	0	0.992	0.000	0.008	0.000
**43**	**50 dpi**	497	436	404	0	31	1	0.927	0.000	0.071	0.002
**44**	**50 dpi**	789	676	628	0	42	6	0.929	0.000	0.062	0.009
**45**	**50 dpi**	630	517	509	0	8	0	0.985	0.000	0.015	0.000
**46**	**50 dpi**	657	563	533	0	29	1	0.947	0.000	0.052	0.002
**47**	**50 dpi**	625	576	31	1	543	1	0.054	0.002	0.943	0.002
**48**	**50 dpi**	732	619	527	4	77	11	0.851	0.006	0.124	0.018
**49**	**Inoculum**	435	375	120	84	83	88	0.320	0.224	0.221	0.235
