# Supplementary material for: Modelling the Evolutionary Dynamics of Viruses within Their Hosts: A Case Study Using High-Throughput Sequencing
Source: PLoS Pathog. 2012 Apr 19;8(4):e1002654. doi: 10.1371/journal.ppat.1002654 (PMC3330117; doi:10.1371/journal.ppat.1002654)
Supplement: Table S2 — Estimation of Ne during plant colonization. (PDF) [file ppat.1002654.s003.pdf]

Table S2. Estimation of N_e during plant colonization

	Initial populations (15 dpi)	Final populations (50 dpi)
H_t [a]	0.565	0.278
H_S [b]	0.366	0.100
F_{ST}	0.352	0.640
N_e	2.249 (1.31 – 3.39) [c]	

[a] genotypic diversity assuming that all plants belong to a single population.

[b] average genotypic diversity within each plant.

[c] The 95% confidence interval was estimated by boostrapping over plants.
