# Supplementary material for: Modelling the Evolutionary Dynamics of Viruses within Their Hosts: A Case Study Using High-Throughput Sequencing
Source: PLoS Pathog. 2012 Apr 19;8(4):e1002654. doi: 10.1371/journal.ppat.1002654 (PMC3330117; doi:10.1371/journal.ppat.1002654)
Supplement: Text S1 — Note on model identifiability and on likelihood-based inference. (PDF) [file ppat.1002654.s004.pdf]

Text S1. Note on model identifiability and on likelihood-based inference.

Conditionally to initial inoculation frequencies, the random vectors $N^p(t_p)$, $p=1,...,40$ (8 plants x 5 sampling dates) of virus sequences obtained by HTS for each plant *p* at its specific time sampling t_p were independent and followed Dirichlet multinomial (DM) distributions. To write down the likelihood of our experiment, we first recall that the probability mass function of a $DM\left(\lambda, N_{tot}, \theta\right)$ distribution has the form

$$\Pr(N^p = y \mid \lambda, N_{tot}, \theta) = p(y \mid \lambda, N_{tot}, \theta) = \frac{N_{tot}!\Gamma(\theta)}{\Gamma(N_{tot}+\theta)} \prod_{i=1}^{4} \frac{\Gamma\left(y_i + \theta\lambda_i\right)}{y_i!\Gamma(\theta\lambda_i)}$$

where $y=(y_1,...,y_4),\ \lambda=(\lambda_1,...,\lambda_4),\ and\ N_{tot}=\sum_{i=1}^{4} y_i$.

Next, let us describe the set of parameters underlying our statistical model. Since we had five sampling dates, i.e. $t_p \in \{6,10,15,24,35\} = \{\tau_s\ ; s=1,...,5\}$, the set of parameters amounted to $\theta^s = \theta(\tau_s)$, and $\lambda^s = \lambda(\tau_s) = (\lambda_1^s,...,\lambda_4^s)$, $s=1,...,5$. Therefore, as $\sum_{i=1}^{4} \lambda_i^s = 1,\ s=1,...,5$, the full statistical model included 20 (=5+3x5) parameters. Actually, the 15 free parameters λ_i^s depended themselves on the 22 (=4+12+1+1+4) unknown parameters r_i, $\beta_{i,j}, 1 \le i \ne j \le 4, K, \mu$ (recall that $\mu_{i,j} = f_{i,j}(\mu)$) and initial conditions $V_i(0)$ of ODE System 1. Consequently, statistical identification of our statistical model would require at least 7 (=22-15) supplementary constraints on the set of parameters of ODE System 1.

Since we dealt only with virus variant frequencies, the time scale (*i.e.* derivatives could be defined up to a constant), the carrying capacity *K* and the number (or size) of virus variants were immaterial at the level of our observations and therefore could be “normalized”. *K* was arbitrarily set to 10^6 and $\sum_{i=1}^{4} r_i = 4$. The mutation rate μ was set to 10^{-5} and, for the initial

values $V(0)=V_{tot}^{inoc}\times(\lambda_1(0),...,\lambda_4(0))$ of ODE System 1, V_{tot}^{inoc} was set 100 whereas $(\lambda_1(0),...,\lambda_4(0))$=(0.32, 0.22, 0.22, 0.24) corresponded to the observed frequencies of virus variants in the inoculum.

Consequently, the remaining set of 20 parameters was now formally identifiable via the following log-likelihood function (withdrawing the indices of θ^s, r_i, and $\beta_{i,j}$)

$$l\,(N^p(t_p), p=1,...,40\,|\,\theta, r, \beta)=\sum_{p=1}^{40}\ln(\,p(N^p(t_p)\,|\,\lambda(t_p\,|\,r,\beta), N_{tot}^p(t_p), \theta(t_p)\,)$$

Additionally, we investigated the sensitivity of the model best supported by the data (Table 1: $\mathfrak{M}_{\mathrm{D_1xC_2}}$). We first analysed the model sensitivity to μ and V_{tot}^{inoc}. Sixteen cases, combining four values (10^{-6}, 10^{-5}, 10^{-4}, 10^{-3}) of μ with four values (10, 100, 1000, 10^4) of V_{tot}^{inoc} were analysed by iterating the same statistical procedure. The percentage of variations of the parameter estimates were all < 5%. We next investigated the model sensitivity to a 20% random fluctuation of the initial frequencies of the variants in the inoculum $(\lambda_1(0),...,\lambda_4(0))$=(0.32, 0.22, 0.22, 0.24). The percentage of variations of the parameter estimates were also < 5% except for θ^1 which reached 15%.
