# Supplementary material for: Modelling the Evolutionary Dynamics of Viruses within Their Hosts: A Case Study Using High-Throughput Sequencing
Source: PLoS Pathog. 2012 Apr 19;8(4):e1002654. doi: 10.1371/journal.ppat.1002654 (PMC3330117; doi:10.1371/journal.ppat.1002654)
Supplement: Text S2 — Mathematical analysis of the Lotka-Volterra model with mutation. (PDF) [file ppat.1002654.s005.pdf]

Text S2. Mathematical Supplement on the Lotka-Volterra model with mutation

In this supplement we give an explicit form of the solution and a proof of the existence of a stable positive equilibria for the Lotka-Volterra model with mutation for the particular interaction $\beta_{i,j} = \frac{r_j}{r_i}$

$$\frac{dV_i(t)}{dt} = r_i V_i \left[1 - \frac{1}{K}\left(V_i + \sum_{j=1, j\neq i}^{4} \beta_{i,j} V_j\right)\right] + \sum_{j=1}^{4} \mu_{ij}(V_j - V_i). \tag{1}$$

We also compute the equilibria for a simplified matrix of mutation rates. As a preliminary we investigate the behaviour of the total population $N(t) = \sum_{i=1}^{4} V_i$.

Behaviour of the total population

Let us first derive the equation satisfied by $N(t)$. To this end, let us introduce the quantities $\alpha(t) := \sum_{i=1}^{4} r_i V_i$, $r_{min} := \min\{r_1, r_2, r_3, r_4\}$ and $r_{max} := \max\{r_1, r_2, r_3, r_4\}$. Then by summing all the equations satisfied by V_i and rearranging the terms we obtain the equation

$$\frac{dN}{dt} = \alpha(t)\left(1 - \frac{N}{K}\right). \tag{2}$$

Therefore one can see that the total population N follows a form of logistic equation. Now let us introduce the functions N_{min} and N_{max} which are respectively the solution of the logistic equations

$$\frac{dN_{min}}{dt} = r_{min} N_{min}\left(1 - \frac{N_{min}}{K}\right), \tag{3}$$

$$N_{min}(0) = N(0), \tag{4}$$

$$\frac{dN_{max}}{dt} = r_{max} N_{max}\left(1 - \frac{N_{max}}{K}\right), \tag{5}$$

$$N_{max}(0) = N(0). \tag{6}$$

Since $r_{min}N(t) \leq \alpha(t) \leq r_{max}N(t)$, one can check that for all times

$$\min\{N_{min}(t), N_{max}(t)\} \leq N(t) \leq \max\{N_{min}(t), N_{max}(t)\}.$$

Hence, $N(t)$ converge to the carrying capacity K exponentially fast (i.e $|K - N| \sim e^{-tr_{min}}$).

Existence of a unique equilibria

Let us first rewrite the system of equation in a more convenient way. Let us define the quantities

$$\alpha(t) = \sum_{i=1}^{4} r_i V_i \quad \text{and} \quad \mu_i = \sum_{j=1}^{4} \mu_{ij}$$

and the following matrix:

$$A(\alpha(t)) := \begin{pmatrix} (r_1 - \frac{\alpha(t)}{K}) - \mu_1 + \mu_{11} & & \mu_{ij} \\ & \ddots & \\ \mu_{ij} & & (r_4 - \frac{\alpha(t)}{K}) - \mu_4 + \mu_{44} \end{pmatrix}.$$

With this notation the system of equation (1) rewrites

$$\frac{dV}{dt} = A(\alpha(t))V \tag{7}$$

and a stationary equilibria $\bar{V}$ for the system (7) will satisfy the following equations:

$$A(\alpha)\bar{V} = 0, \tag{8}$$

$$\alpha = \sum_{i}^{4} r_i \bar{V}_i. \tag{9}$$

By introducing the two matrices

$$M := \begin{pmatrix} -\mu_1 + \mu_{11} & & \mu_{ij} \\ & \ddots & \\ \mu_{ij} & & -\mu_4 + \mu_{44} \end{pmatrix} \quad \text{and} \quad R := \begin{pmatrix} r_1 & & 0 \\ & \ddots & \\ 0 & & r_4 \end{pmatrix},$$

the matrix $A(\alpha)$ rewrites $A(\alpha) = \left(R - \left(\frac{\alpha}{K}\right) Id + M\right)$ and one can see that a stationary equilibria $\bar{V}$ must satisfy the equation :

$$(M + R)\bar{V} = \left(\frac{\alpha}{K}\right)\bar{V}. \tag{10}$$

Lemma 0.1 *If $\bar{V}$ is a non negative stationary solution, then either $\bar{V} \equiv 0$ or $\bar{V} > 0$ (i.e $\forall i$, $\bar{V}_i > 0$).*

Proof:

First, observe that 0 is a solution of the problem (8). Now, let us assume there exists a non negative stationary solution $\bar{V} \not\equiv 0$. Then we must have $\bar{V}_j > 0$ for all j. Indeed, assume that $\bar{V}_i = 0$ for some i, then from (10) we have

$$(M + R\bar{V})_i = \left(\frac{\alpha}{K}\right)\bar{V}_i = 0.$$

Therefore we get the contradiction

$$0 = r_i \bar{V}_i + \sum_{j=1}^{4} \mu_{ij}(\bar{V}_j - \bar{V}_i) = \sum_{j=1}^{4} \mu_{ij} \bar{V}_j > 0.$$

Hence $\bar{V}_j > 0$ for all j.

□

Observe that from the above Lemma and from (10), a non trivial equilibria $\bar{V}$ is always a positive eigenvector of the matrix $M + R$ associated with the eigenvalue $\frac{\alpha}{K}$. We are now in position to prove the existence of a unique positive stationary solution to (10).

Lemma 0.2 *There exists a unique* α *and* $\bar{V}$ *solution to* (8) *and* (9)*. Moreover,* $\bar{V}$ *satisfies* $\sum_{i=1}^{4} \bar{V}_i = K$*.*

Proof:

Let $\bar{\mu} = \sup_{i \in \{1,\dots,4\}} \mu_i$. Since $R + M + \bar{\mu} Id$ is a non negative matrix, by the Perron-Frobenius Theorem there exists a unique principal eigenvalue (ν_p, v_p) with a positive eigenvector v_p, i.e. (ν_p, v_p) satisfies

$$(R + M + \bar{\mu} Id) v_p = \nu_p v_p. \tag{11}$$

Moreover, the eigenspace associated to the eigenvalue ν_p is one dimensional, see [1]. Let us choose $v_p > 0$ so that $\sum_{i=1}^{4} (v_p)_i^2 = 1$. From the equation (11), we deduce that the vector v_p is a positive eigenvector of the matrix $M + R$ associated with the eigenvalue $\lambda_p := (\nu_p - \bar{\mu})$. By construction one can see that λ_p is the unique eigenvalue of the matrix $M + R$ associated with a positive eigenvector. A quick computation shows that $\lambda_p = (\nu_p - \bar{\mu}) > 0$. Indeed, if not we have

$$(R + M) v_p \leq 0.$$

Thus for all $i \in \{1, \dots, 4\}$ we have

$$r_i (v_p)_i + \sum_{j=1}^{4} \mu_{ij} \big((v_p)_j - (v_p)_i \big) \leq 0.$$

Let $(v_p)_{i_0} := \min_{i \in \{1,\dots,4\}} (v_p)_i$ then for $(v_p)_{i_0}$ we have

$$\sum_{j=1}^{4} \mu_{ij} \big((v_p)_j - (v_p)_{i_0} \big) \geq 0$$

and since R is a positive matrix we achieve the contradiction

$$0 < r_{i_0} (v_p)_{i_0} + \sum_{j=1}^{4} \mu_{ij} \big((v_p)_j - (v_p)_{i_0} \big) \leq 0.$$

Now from (10) we deduce that there exists a unique α so that $\frac{\alpha}{K} = \lambda_p$, which is $\alpha = K\lambda_p$. Now let us construct our solution. Note that for any $\lambda \in \mathbb{R}$, the vector λv_p is also a solution to (10) with the eigenvalue λ_p. So to obtain a solution $\bar{V}$ to (8) and (9) we only have to adjust λ in such a way that $\sum_i \lambda r_i (v_p)_i = \alpha$, which corresponds to take

$$\lambda = \frac{K\lambda_p}{\sum_i^4 r_i (v_p)_i}.$$

Note that the solution $(K\lambda_p, \frac{K\lambda_p}{\sum_i^4 r_i(v_p)_i} v_p)$ satisfies

$$\frac{K\lambda_p}{\sum_i^4 r_i (v_p)_i} \sum_{i=1}^4 (v_p)_i = K.$$

Indeed, since v_p is an eigenvector associated with λ_p we have

$$(R + M)v_p = \lambda_p v_p.$$

So we will have

$$\sum_{i=1}^4 r_i (v_p)_i = \sum_{i=1}^4 ((R + M)v_p)_i = \lambda_p \sum_{i=1}^4 (v_p)_i.$$

Since we know that $\lambda_p > 0$, we deduce that

$$\frac{\sum_{i=1}^4 (v_p)_i}{\sum_i^4 r_i (v_p)_i} = \frac{1}{\lambda_p}.$$

Hence,

$$\frac{K\lambda_p}{\sum_i^4 r_i (v_p)_i} \sum_{i=1}^4 (v_p)_i = K.$$

Explicit form of the solution $V(t)$

Here let us derive an explicit formula for the solution $V(t)$ of (1). To this end let us introduce the function

$$v_i(t) := e^{\int_0^t \alpha(s)\, ds} V_i(t)$$

and remark that the v_i satisfy the linear equation

$$\frac{dv_i(t)}{dt} = r_i v_i + \sum_{j=1}^4 \mu_{ij}(v_j - v_i).$$

Thus $v_i(t) := \left(e^{(R+M)t} V(0)\right)_i$ since $v_i(0) = V_i(0)$ and $V_i(t)$ is implicitly given by the formula

$$V_i(t) = e^{-\int_0^t \alpha(s)\, ds} \left(e^{(R+M)t} V(0)\right)_i.$$

Now let us evaluate the term $e^{-\int_0^t \alpha(s)\,ds}$. By differentiating $e^{-\int_0^t \alpha(s)\,ds}$ we have

$$\frac{d}{dt}(e^{\int_0^t \alpha(s)\,ds}) = \alpha(t)e^{\int_0^t \alpha(s)\,ds} = \sum_{j=1}^{4} r_j V_j e^{\int_0^t \alpha(s)\,ds} = \sum_{j=1}^{4} r_j v_j(t).$$

Therefore one has

$$e^{\int_0^t \alpha(s)\,ds} = 1 + \int_0^t \sum_{j=1}^{4} r_j v_j(s)\,ds,$$

which rewrites

$$e^{\int_0^t \alpha(s)\,ds} = 1 + \sum_{j=1}^{4} r_j \int_0^t v_j(s)\,ds = 1 + \sum_{j=1}^{N} r_j \int_0^t (e^{(R+M)s}V(0))_j\,ds.$$

Hence we have

$$V_i(t) = \frac{\left(e^{(R+M)t}V(0)\right)_i}{1 + \sum_{j=1}^4 r_j \int_0^t (e^{(R+M)s}V(0))_j\,ds}.$$

An illuminating example

Let us look deeper on the structure of the steady state. For a general setting it will be very hard to get an analytic formula expressing each density, however one can extract some information for a particular structure of the mutation matrix M. Let us consider M the following matrix of mutation :

$$M := \begin{pmatrix} (1-\mu)^2 - 1 & \mu(1-\mu) & \mu(1-\mu) & \mu^2 \\ \mu(1-\mu) & (1-\mu)^2 - 1 & \mu^2 & \mu(1-\mu) \\ \mu(1-\mu) & \mu^2 & (1-\mu)^2 - 1 & \mu(1-\mu) \\ \mu^2 & \mu(1-\mu) & \mu(1-\mu) & (1-\mu)^2 - 1 \end{pmatrix},$$

where μ is a parameter giving the point mutation rate per replication cycle and per nucleotide. This particular matrix M corresponds to a set of 4 virus variants differing only by one or two substitutions used in this study. For simplicity, we assume that the fittest variant of the system correspond to the first variant.

Now by defining the following two matrices M_{red} and C

$$M_{red} := \begin{pmatrix} -2 & 1 & 1 & 0 \\ 1 & -2 & 0 & 1 \\ 1 & 0 & -2 & 1 \\ 0 & 1 & 1 & -2 \end{pmatrix}, \qquad C := \begin{pmatrix} 0 & 0 & 0 & 1 \\ 0 & 0 & 1 & 0 \\ 0 & 1 & 0 & 0 \\ 1 & 0 & 0 & 0 \end{pmatrix},$$

one can see that $M = \mu M_{red} + \mu^2 C = \mu M_{red} + O(\mu^2)$.

So, as $\mu \sim 10^{-5} << 1$, after neglecting the quadratic terms in μ one have $M \approx \mu M_{red}$. Let us now investigate the steady state solution of the problem (8)-(9) with μM_{red} instead of M. According to the above computation, the stationary solution is given by the formula

$$\frac{K(\lambda_p - 2\mu)}{\sum_i^4 r_i (v_p)_i} v_p,$$

where λ_p and v_p are respectively the principal eigenvalue and positive eigenvector of $R + \mu M_{red} + 2\mu Id$.

An analytic expression of the steady state will then follow from the behaviour of λ_p and v_p with respect to small μ. To this end we first recall the following result:

Theorem 0.3 (Differentiability of the eigenvalues) *Let λ_1 be an algebraically simple eigenvalue of a $n \times n$ symmetric matrix A and let C be a another $n \times n$ matrix.Then for $\epsilon > 0$ small enough the matrix $A(\epsilon) := A + \epsilon C$ has a unique eigenvalue $\lambda_1(\epsilon)$ of the form*

$$\lambda_1(\epsilon) = \lambda_1 + \epsilon \frac{{}^t v_1 C v_1}{v_1 \bullet v_1} + O(\epsilon^2),$$

where v_1 satisfies $Av_1 = \lambda_1 v_1$ and $u \bullet v$ denotes the standard scalar product between two vectors. Moreover the eigenvector $v_1(\epsilon)$ satisfies

$$(i) \quad v_1(\epsilon) \bullet v_1(\epsilon) = 1,$$
$$(ii) \quad v_1(\epsilon) = v_1 + \epsilon \sum_{i=2}^{n} \frac{{}^t v_i C v_1}{\lambda_1 - \lambda_i} \Big(v_i - v_1 (v_1 \bullet v_i) \Big) + O(\epsilon^2).$$

Applying the above theorem in our example it follows that the principal eigenvalue $\lambda_p(\mu)$ of $R + \mu M_{red}$ and its corresponding principal eigenvector $v_p(\mu)$ are given by

$$\lambda_p(\mu) = \lambda_p(R) + \mu \frac{{}^t v_1 M_{red} v_1}{v_1 \bullet v_1} + O(\mu^2),$$
$$v_p(\mu) = v_p + \mu \sum_{i \neq i_0}^{4} \frac{{}^t v_i M_{red} v_p}{\lambda_p - \lambda_i} \Big(v_i - v_p (v_p \bullet v_i) \Big) + O(\mu^2),$$

where v_p is the principal eigenvector associated to $\lambda_p(R)$. Since R is diagonal, we have $\lambda_i = r_i$ and $v_i = e_i$ the corresponding unit vector.
Therefore $\lambda_p(R) = r_{max} := max\{r_1, r_2, r_3, r_4\} = r_1$ and $v_1 = e_1$. So we have

$$\lambda_p(\mu) = r_1 - 2\mu + O(\mu^2),$$
$$v_p(\mu) = e_1 + \mu \sum_{i=2}^{3} \frac{1}{r_1 - r_i} e_i + O(\mu^2),$$

since ${}^t e_1 M_{red} e_4 = 0$. Note that the above formulas are only valid for $\mu << \min_{i \neq 1}\{r_1 - r_i\}$.

Going back to the formula of the steady states and plugging the above asymptotic formulas for $\lambda_p(\mu)$ and $v_p(\mu)$ we end up with

$$\bar{V} = \frac{K(r_1 - 2\mu)}{r_1 + \mu \sum_{i=2}^{3} \frac{r_i}{r_1 - r_i}} \left(e_1 + \mu \sum_{i=2}^{3} \frac{1}{r_1 - r_i} e_i \right) + O(\mu^2).$$

Now for $\mu << \frac{1}{\sum_{i=2}^{3} \frac{r_i}{r_1 - r_i}}$ using a Taylor expansion one has

$$\frac{1}{r_1 + \mu \sum_{i=2}^{3} \frac{r_i}{r_1 - r_i}} = \frac{1}{r_1} \left(1 - \frac{\mu \sum_{i=2}^{3} \frac{r_i}{r_1 - r_i}}{r_1} + O(\mu^2) \right)$$

and we can see that

$$\bar{V} = K \left[1 - \frac{\mu}{r_1} \left(2 + \sum_{i=2}^{3} \frac{r_i}{r_1 - r_i} \right) \right] e_1 + K\mu \sum_{i=2}^{3} \frac{1}{r_1 - r_i} e_i + O(\mu^2),$$

which rewrites

$$\bar{V} = \begin{pmatrix} K \\ 0 \\ 0 \\ 0 \end{pmatrix} + \mu K \begin{pmatrix} -\frac{1}{r_1} \left(2 + \frac{r_2}{r_1 - r_2} + \frac{r_3}{r_1 - r_3} \right) \\ \frac{1}{r_1 - r_2} \\ \frac{1}{r_1 - r_3} \\ 0 \end{pmatrix} + O(\mu^2).$$

As a first consequence of this formulas, on can see that the variants which are not produced by mutations by the fittest variant are only appearing in small quantities of order $O(\mu^2)$.

References

[1] Zeidler, E. *Nonlinear Functional Analysis and Its Applications*, Springer-Verlag Berlin and Heidelberg GmbH & Co. K, 1986, ISBN: 3540909141.
